# Zinc Oxide Nanoparticles (ZnO NPs) and N-Methylol Dimethyl Phosphonopropion Amide (MDPA) System for Flame Retardant Cotton Fabrics

**DOI:** 10.3390/polym14163414

**Published:** 2022-08-21

**Authors:** Asif Javed, Jakub Wiener, Jana Saskova, Jana Müllerová

**Affiliations:** 1 Department of Material Engineering, Faculty of Textile Engineering, Technical University of Liberec, Studentska 1402/2, 461 17 Liberec, Czech Republic; 2 Department of Nanochemistry, Institute for Nanomaterials, Advanced Technologies and Innovation, Technical University of Liberec, Studentska 1402/2, 461 17 Liberec, Czech Republic

**Keywords:** flame retardants, antibacterial, ZnO, nanoparticles, metal oxides

## Abstract

The aim of the present research work was to develop halogen and formaldehyde-free, durable flame retardant fabric along with multifunctional properties and to find the optimal conditions and parameters. In this research, zinc oxide nanoparticles (ZnO NPs) were grown onto 100% cotton fabric using the sonochemical method. Zinc acetate dihydrate (Zn(CH_3_COO)_2_·2H_2_O) and sodium hydroxide (NaOH) were used as precursors. After ZnO NPs growth, N-Methylol dimethylphosphonopropionamide (MDPA) flame retardant was applied in the presence of 1, 2, 3, 4-butanetetracarboxylic acid (BTCA) as cross-linkers using the conventional pad–dry–cure method. Induced coupled plasma atomic emission spectroscopy (ICP-AES) was used to determine the deposited amount of Zn and phosphorous (P) contents. Scanning electron microscopy (SEM), X-ray powder diffraction (XRD), and Fourier-transform infrared spectroscopy (FTIR) were employed to determine the surface morphology and characterization of the developed samples. Furthermore, the thermal degradation of the untreated and treated samples was investigated by thermogravimetric analysis (TGA). Furthermore, the vertical flame retardant test, limiting oxygen index (LOI), ultraviolet protection factor (UPF), and antibacterial activity of samples were examined. The developed samples showed excellent results for flame retardancy (i.e., 39 mm char length, 0 s after flame time, 0 s after glow time), 32.2 LOI, 143.76 UPF, and 100% antibacterial activity.

## 1. Introduction

Cotton fiber is one of the most plentifully used fibers all over the world. It is comfortable, cozy, and breathable when used in garment form [1,2,3]. However, it is one of the most combustible fabrics and is very susceptive to thermal decomposition. It exhibits a very low oxygen-limiting index and is a highly flammable fiber. It burns very quickly with a hot flame and little sparks [4,5]. Moreover, with flammability and combustibility, cotton fabric provides an indigent defense to human skin against UV radiation and bacterial growth. Therefore, these are fundamental problems regarding cotton fabric, limiting its use in industrial work wear, housing, and technical applications [6,7].

Flame retardant treatment on textile fabrics has gained significant importance because flame retardant fabrics can be used as safety work wear in industry, firefighting, hospitals, and in household upholstery [8,9]. Various chemical applications are involved in producing flame retardant fabrics, but most of the flame retardant chemicals contain halogen compounds that are not environmentally friendly [10,11,12]. Phosphorous-based durable flame retardant chemicals are alternative to halogen compounds. These phosphorous-based compounds are environmentally friendly and economical for cotton textile application [13]. N-methylol dimethyl phosphonopropion amide (MDPA) is one of the most promising flame retardant compounds due to its durability, low toxicity, environmentally friendly nature, and convenient application. When it is applied along with a cross-linker onto the cotton fabric, it develops a covalent bond with a hydroxyl group of cotton cellulose, enhancing its durability [14,15,16].

Nanotechnology is another important field that has been utilized successfully and efficiently in the industry to achieve desired fruitful results. Nanomaterials in the textile industry have earned great importance due to their multipurpose uses [17]. Nanomaterials in the form of nanoparticles are being used in the textile industry for antibacterial textiles [18], UV protection, increased flame retardancy, self-cleaning, electromagnetic shielding (EMI), conductive textiles, etc. [19,20,21]. Among the nanoparticles used in industry, metal oxides are of great importance because of their multipurpose properties [22]. Zinc Oxide (ZnO) is one of the most versatile inorganic metal oxides. It is an n-type semiconductor, white in color, with a high refractive index and a wide band gap of 3.37 ev [23,24]. Along with UV protection, antibacterial, and self-cleaning properties, zinc oxide nanoparticles (ZnO NPs) are being used in flame retardant coating [25,26]. ZnO NPs are used as co-catalysts in flame retardant finishings and are very effective in char formation during the burning of fabric [12]. Researchers showed that ZnO NPs, when used as co-catalysts, increase the flame retardancy of cotton fabric by increasing its thermal stability, as well as increasing the durability of the flame retardant [27,28,29]. Various techniques are being used for in situ synthesis of ZnO NPs onto fabric, such as hydrothermal, solvochemical, sol-gel, precipitation method, sonochemical, microwave irradiation method, etc. [30,31].

The main challenges in the flame retardant/ZnO NPs system are the use of formaldehyde-free cross-linker, homogeneous, and stable deposition of ZnO NPs. BTCA is a formaldehyde-free cross-linker that can be used for cotton flame retardant systems [32,33]. For ZnO NPs, Javed et al. reported that sonochemical is an advanced and economical method for the in situ synthesis of ZnO NPs onto cotton fabric. This technique controls the nanoparticle size without affecting the strength of the fabric. It also allows ultrasonic waves to disperse and deposit the nanoparticles onto the fabric more stably, homogeneously, and evenly [34].

In this research, ZnO NPs were in situ synthesized onto cotton fabric using the ultrasonic irradiation method. After that, MDPA flame retardant in the presence of formaldehyde-free cross-linker BTCA was applied onto the cotton fabric by the pad–dry–cure method. The main goal of the present work was to determine the optimized parameters for in situ sonochemical synthesis of ZnO NPs and investigate the role of ZnO NPs in flame retardant finishing and their influence on the functional properties. 

To the best of the authors’ knowledge, this is the first study on flame retardant application in combination with MDPA along with formaldehyde-free cross-linkers and ZnO NPs using the sonochemical method. The finding of this study could be beneficial for flame retardant application in safety textiles (welding work wear, electrical work wear, industrial work wear, etc.).

## 2. Materials and Methods

### 2.1. Materials

The 100 percent cotton fabric with a plain weave texture, 155 g/m^2^ density, 52 ends/inch, 28 picks/inch, and 20 tex warp count, 20 tex filling count, was acquired from the Technical University of Liberec, Czech Republic. Citric acid C_6_H_8_O_7_, zinc acetate dihydrate (Zn(CH_3_COO)_2_·2H_2_O), sodium hydroxide (NaOH), sodium hypophosphite (SHP), and 1, 2, 3, 4-butanetetracarboxylic acid (BTCA) chemical reagents were procured from Merk, Prague, Czech Republic. N-Methylol dimethylphosphonopropionamide (MDPA) was obtained from the Huntsman Corporation. Acramin SW acrylic-based binder was obtained from Tanatex Chemicals, The Netherlands. All the obtained chemical reagents were of analytical grade and utilized as purchased without further purification.

### 2.2. Surface Functionalization of Cellulose

To obtain maximum adherence of ZnO NPs and MDPA on the cellulosic structure of the cotton fabric, the cotton fabric was pretreated with a 0.5% aqueous solution of citric acid in the presence of 0.5% sodium hypophosphite as a catalyst for cellulosic surface functionalization. As the citric acid and cotton fibers were added to deionized water, both were ionized, as shown in Equations (1) and (2). In a further reaction, carboxylic groups of citric acid were easily attached to the hydroxyl groups on the cotton fabric, as shown in Equation (3) [20].

(1) $$C_{6}H_{8}O_{7}+{\;H}_{2}O\;↔C_{6}H_{7}O_{7}^{-}+{\;H}_{3}O^{+},$$

(2) $$\mathrm{Cellulose}-\mathrm{OH}+{\;H}_{2}O\;↔\mathrm{Cellulose}-O+{\;H}_{3}O^{+},$$

(3) $$C_{6}H_{7}O_{7}^{-}+\mathrm{Cellulose}-\mathrm{OH}+H_{2}O\;↔\;\mathrm{Cellulose}-\mathrm{CA}+{\;H}_{3}O^{+}.$$

### 2.3. In-Situ Sonochemical Synthesis of ZnO NPs on Cotton Fabric

ZnO NPs were synthesized and stabilized onto the cotton fabric concomitantly by hydrolysis of zinc acetate dihydrate (Zn(CH_3_COO)_2_·2H_2_O) and sodium hydroxide (NaOH) in deionized water. The precursors, zinc acetate dihydrate (Zn(CH_3_COO)_2_·2H_2_O) (0.05 M, 0.1 M, 0.15 M) and sodium hydroxide (NaOH) (0.1 M, 0.2 M, 0.3 M) with different molar concentrations, were dissolved separately in deionized water under vigorous magnetic stirring (300 rpm) conditions. After that, the cotton fabric piece was dipped into the zinc acetate dihydrate solution for 10 min under vigorous magnetic stirring (300 rpm). After 10 min, the NaOH solution was poured dropwise into that solution at ambient temperature and under vigorous magnetic stirring (300 rpm). For absolute completion of the reaction mechanism, the obtained solution containing the immersed cotton fabric piece was sonicated for different sonication times (30 min, 60 min, 90 min, and 120 min). The Branson sonication probe (20 kHz, 50% efficiency, 150 W) was utilized in this experimental procedure. The reaction temperature was maintained at 80 °C by utilizing a hot plate. Then, the treated fabric pieces were washed thoroughly with deionized water to remove any impurities. Eventually, the obtained fabric pieces were placed in an air oven at 90 °C for 120 min. In order to compare the sonochemical process and to accentuate the critical influence of ultrasound irradiation waves, one sample was developed using a conventional magnetic stirring method using the same precursor concentrations (0.1 M Zn(CH_3_COO)_2_·2H_2_O, 0.3 M NaOH) and temperatures (80 °C) as the optimized sample, under vigorous magnetic stirring (300 rpm) for 90 min. In this research work, this sample was named sample A. Equations (4)–(6) show the proposed mechanism of ZnO NPs synthesis on the cotton fabric.

(4) $$\mathrm{Zn}{\left({\mathrm{CH}}_{3}\mathrm{COO}\right)}_{2}\cdot 2H_{2}O+2\mathrm{NaOH}\to 2{\mathrm{CH}}_{3}\mathrm{COONa}+{\mathrm{Zn}}^{+}+2{\mathrm{OH}}^{-}+2H_{2},$$

(5) $${\mathrm{Zn}}^{+}+2{\mathrm{OH}}^{-}\;\to \mathrm{Zn}{\left(\mathrm{OH}\right)}_{2},$$

(6) $$\mathrm{Zn}{\left(\mathrm{OH}\right)}_{2}\to \mathrm{ZnO}+{\;H}_{2}O.$$

### 2.4. MDPA Application

MDPA application was performed with the help of a laboratory padder (Werner Mathis AG Switzerland) at 80% wet pick up. The bath formulation used 300 g/L MDPA, 60 g/L BTCA crosslinker, 50 g/L SHP catalyst, and 5 g/L acramin SW binder. Various preliminary trials were conducted to determine the best compatible concentrations of MDPA and BTCA with optimized ZnO NPs loaded samples. ZnO NPs loaded samples were impregnated in MDPA and BTCA solution, padded and dried at 110 °C for 3 min, and cured at 150 °C for 2 min. In order to determine the crucial role of ZnO NPs in flame retardancy, a cotton fabric sample was treated with MDPA and BTCA without ZnO NPs treatment. In this research work, that sample was named sample B.

**Figure 1:**
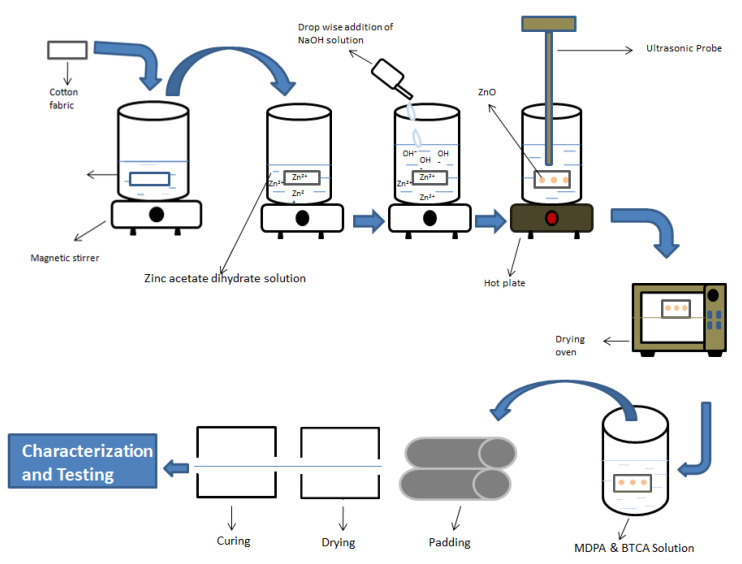
Schematic diagram for surface functionalization of cellulose, in situ synthesis of ZnO NPs on the cotton fabric, and MDPA application.

Table 1 show the complete experimental design for the in situ synthesis of ZnO NPs on the cotton fabric and MDPA application. Figure 1 show the schematic diagram for the surface functionalization of cellulose, in situ synthesis of ZnO NPs on the cotton fabric, and MDPA application. Table 2 show the results for flammability and functional properties.

### 2.5. Characterization and Testing of Functional Properties

The induced coupled plasma atomic emission spectrometer (ICP AES, Optima7300 DV, Perkin-Elmer Corporation, Waltham, MA, USA) was utilized to analyze the zinc (Zn) and phosphorous (P) content. The developed fabric sample weighing 0.1 g was treated with 8 mL of concentrated nitric acid (HNO_3_) (65%) until the fabric wholly dissolved. Then, the obtained solution was shifted to a volumetric flask of 100 mL capacity, and finally, dilution was carried out with deionized water.

The *add-on%* (uptake) was calculated according to Equation (7) and tabulated in Table 1.

(7) $$Add\;on\%=\frac{wf-wi}{wi}\times 100.$$

In Equation (7), *wf* is the final weight of the developed sample and *wi* is the initial weight of the untreated sample.

The surface of the pristine cotton and developed samples was visualized using a Quanta 200 FEG scanning electron microscope (SEM) (FEI Company, Hillsboro, OR, USA).

The particle size of the synthesized ZnO NPs was examined by employing dynamic light scattering (DLS) technology using the Malvern zeta sizer (Malvern Panalytical Ltd., Malvern, UK). The ZnO NPs-coated fabric was dissolved in concentrated HNO_3_ (65%). After that, the obtained solution was diluted with deionized water and then centrifuged to obtain ZnO NPs. The obtained ZnO NPs were dispersed in deionized water with the help of an ultrasonic probe. Eventually, the DLS technique was employed.

The XRD patterns were measured using an X-ray diffraction system (Powder X-ray diffraction system, ARL, X TRA, Thermo Scientific, Waltham, MA, USA). The measurements were recorded in the range of diffraction angle 2Ɵ = 10°–70°, with step size 0.02, a scan rate of 2 [°/m], and 0.6 integration duration. The average nano crystallite size was calculated by employing the Scherrer Equation (8).

(8) $$d=\frac{Kʎ}{\beta cosƟ}.$$

In Equation (8), *d* is the nanocrystallite size, *K* is the scherrer constant (0.89), ʎ is the X-ray wavelength, *β* is the full-width at half maximum of the peak, and Ɵ is the Bragg diffraction angle.

Fourier transform infrared spectroscopy (FTIR) was employed on the developed samples and pristine cotton to investigate the surface chemical structure. The measurements were performed at room temperature with the help of a Perkin Elmer spectrometer equipped with Thermo Scientific Nicolet IS50 FT-IR USA attenuated total reflectance (ATR) technology. The spectra were recorded in the range of 4000 to 400 cm^−1^ using ZnSe crystal at a resolution of 4 cm^−1^ with 32 scans.

The thermal stability of the developed and untreated samples was examined with the help of thermogravimetric analysis (TGA) using a TGA/SDTA 851 METLER TOLEDO analyzer. The untreated and developed samples were subjected to heat in a synthetic air atmosphere from 30 °C to 700 °C with a 10 °C/min heating rate. Finally, the weight loss percentage of the samples was measured.

To evaluate the flammability of the untreated cotton samples and developed samples, a vertical flame test (ASTM 6413-2015) was employed.

The LOI values were recorded for untreated and developed samples according to the standard test method ASTMD 2863-97. In this method, the sample is ignited with a combustible flame in an oxygen/nitrogen environment. Then, the oxygen concentration in the oxygen/nitrogen environment is decreased until the flame is extinguished. The minimum concentration of oxygen which supports the combustion is recorded. LOI is expressed as a volume percentage and calculated according to the following Equation (9).

(9) $$LOI=\left(100\times O_{2}\right)\left(O_{2}+N_{2}\right).$$

Pristine cotton samples and treated samples were analyzed for their UV protective properties on a Varian CARY 1E UV/VIS spectrophotometer equipped with a DRA-CA-301 integration sphere and solar screen software. The samples were measured in the UV range of 280 nm to 400 nm. The transmittance measurements and calculations of the UPF were carried out in accordance with the AATCC TM 183 standard. The UPF value was calculated according to Equation (10).

(10) $$\mathrm{UPF}=\frac{\sum_{280\mathrm{nm}}^{400\mathrm{nm}}E_{\lambda }S_{\lambda }\Delta_{\lambda }}{\sum_{280\mathrm{nm}}^{400\mathrm{nm}}E_{\lambda }S_{\lambda }T_{\lambda }\Delta_{\lambda }}.$$

E_λ_ is the solar spectral irradiance, S_λ_ is the relative erythemal spectral response, Δ_λ_ is the measured wavelength interval in nanometers, and T_λ_ is the average spectral transmittance from the sample.

The quantitative method AATCC 100-2012 was used to analyze the antibacterial performances of the samples. According to this standard, 1 mL of bacterial inocula was taken in a conical flask, and fabric pieces (4.8 ± 0.1 cm diameter) were added to that flask and allowed to remain in contact with the bacterial inocula for 24 h. After that, the solution was subjected to serial dilution up to 10^−7^ in nutrient broth. Then, the 0.1 mL of the dilution was transferred to an agar plate and finally incubated for the duration of 24 h at a temperature of 37 °C. The no. of bacterial colonies that appeared was counted. The bacterial reduction % was calculated according to the following Equation (11).

(11) $$R\%=\frac{A-B}{A}\times 100$$

where R is the bacterial reduction %, A is the no. of bacterial colonies that appeared from the untreated sample, and B is the no. of bacterial colonies that appeared from the treated sample.

The home laundering washing durability of the treated samples was examined as per the ISO 105-CO6 standard. Each wash cycle of this method is equal to five home laundering. The treated samples were washed at 50 °C for 45 min in the presence of 4 g/L washing detergent. Finally, the washed samples were rinsed and then dried at 80 °C. Eventually, the washed samples were investigated for their functional properties.

## 3. Results

All the results present in this research study are the mean of five replications.

### 3.1. Content Analysis

ICP-AES technique was employed to analyze the Zn and P contents of the developed samples and tabulated in Table 1. Zn contents analysis was conducted to decide the most productive and optimized sonication time and concentrations of chemical reagents. It can be seen from Table 1 that with increased sonication time up to 90 min synthesis, the mass of Zn contents also increased. However, after being synthesized for 90 min, the mass of Zn contents decreased. After a critical time of 90 min, ultrasonic waves might lead to the removal the ZnO NPs from the fabric. The maximum synthesized mass of Zn contents was achieved for sample 23, which exhibited Zn contents of 13.14%. Therefore, the optimal sonication time for this experiment is 90 min, and the optimal concentrations of the reagents are 0.1 M Zn(CH_3_COO)_2_·2H_2_O and 0.3 M NaOH. It is evident from Table 1 that the sonication time and concentrations of chemical reagents have a significant effect on the mass of Zn contents synthesized.

### 3.2. SEM Analysis

**Figure 2:**
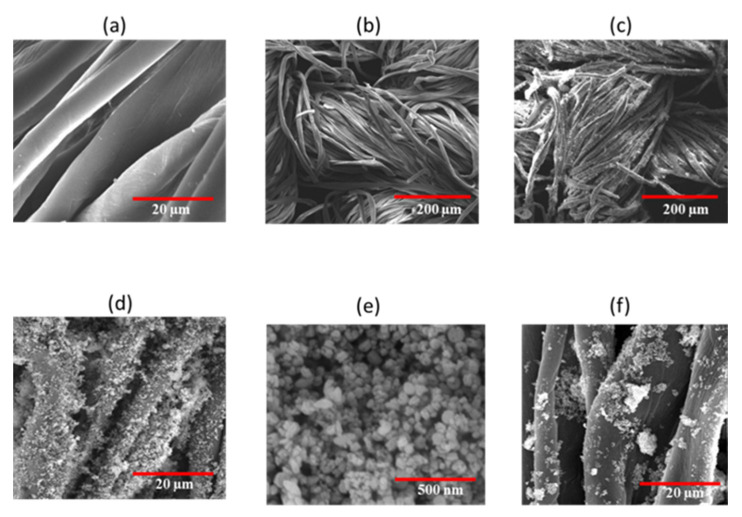
SEM images (**a**,**b**) pristine cotton, (**c**–**e**) sample 23, and (**f**) sample A.

SEM Images were measured to investigate the surface morphology of the pristine cotton fabric and ZnO NPs-coated samples. It can be seen from Figure 2a,b that pristine cotton has a clean and smooth surface. Figure 2c,d show the SEM images for optimized sample 23, which reveals that after optimized sonochemical treatment, ZnO NPs are spread onto the cotton fabric surface homogeneously, finely, and evenly. Figure 2c,d show that the surface of the fabric is entirely covered by the ZnO NPs. The deposition of ZnO NPs onto the cotton fabric surface results from attractive forces between cellulosic functional groups and ZnO NPs [35]. SEM images show that deposition of ZnO NPs created roughness on the surface of fibers. The Figure 2f SEM image for sample A shows that there is a deposition of ZnO NPs onto the cotton fabric surface after the conventional magnetic stirring method, but as compared to the sonochemical method, the ZnO NPs are not spread smoothly, finely, and homogeneously. Figure 2e show the SEM image for the optimized sample 23 at high resolution, showing that ZnO particles are deposited onto the cotton surface at a nanoscale with narrow size distribution. Moreover, Figure 2e reveal that most of the ZnO NPs have a round and spherical shape.

### 3.3. Particle Size

**Figure 3:**
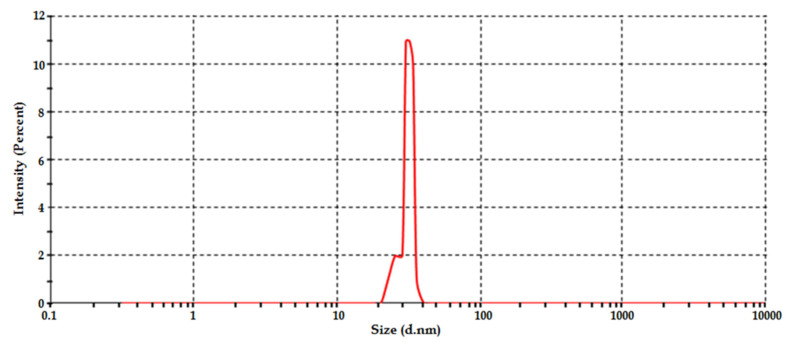
Particle size distribution Sample 23.

Figure 3 show the particle size distribution of sonochemical in situ synthesized ZnO NPs (optimized sample 23). It can be seen from the figure that nanoparticle size distribution is uni-modal, with an average particle size of 30.89 nm. At the nanoscale, the particles show increased surface areas, allowing the nanoparticles to be utilized in many technical applications [36,37].

### 3.4. XRD Analysis

**Figure 4:**
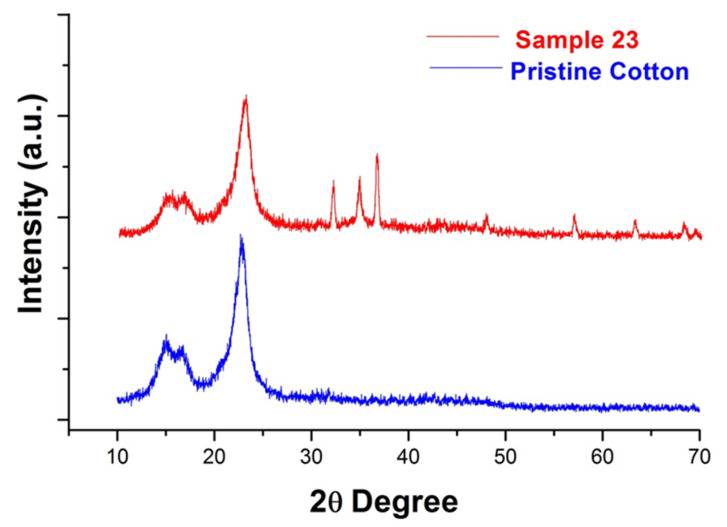
XRD diffractogram of pristine cotton and sample 23.

The XRD diffractograms of pristine cotton fabric and optimized sonochemically treated sample 23 are presented in Figure 4. It is obvious from Figure 4 that the pristine cotton fabric only has the characteristic peaks of cellulose (at 2Ɵ = 14.8, 16.5, and 22.7) (JCDPS No. 03-0226) [38]. In comparison, sample 23 has some additional peaks (at 2Ɵ = 32.1, 34.7, 36.5, 47.8, 56.7, 63.1, 68.1, 69.2) in the diffraction planes (100), (002), (101), (102), (110), (103), (200), and (112). These are characteristic peaks of ZnO NPs (as per diffraction standard No. 36-1451 defined by the Joint Committee on powder diffraction standard (JCDPS)) [39]. The additional peaks are evidence of the presence of the crystalline hexagonal wurtzite structure of ZnO NPs [40,41,42]. The peak in the plane (002) has the highest intensity, which shows that the c-axis direction is the main dominant and leading growth direction for ZnO NPS. Moreover, in the case of sample 23, the peak intensity of the cellulose decreased due to ZnO NPs loading. Furthermore, there is no extra peak in the diffractogram of sample 23 other than cellulose and ZnO NPs, which shows the purity of the ZnO NPs. The crystallite size measured by the Scherrer equation for sample 23 was 22.4 nm.

### 3.5. FTIR Analysis

**Figure 5:**
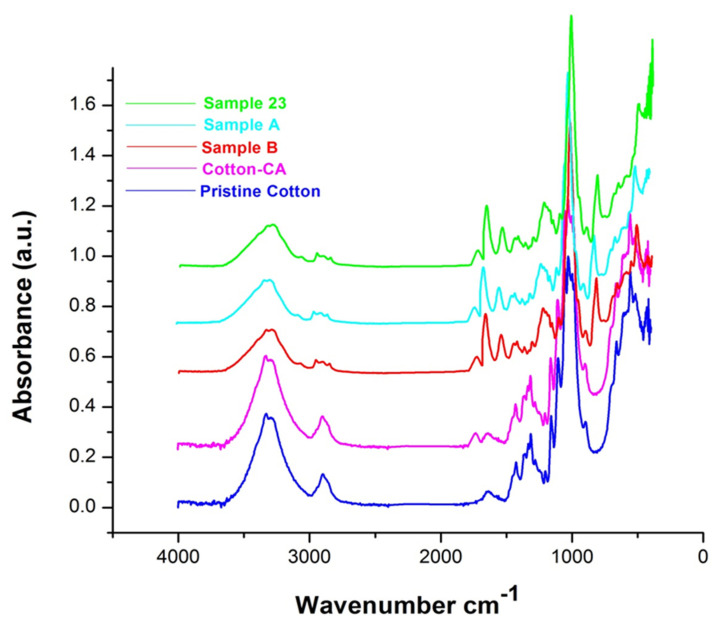
FTIR spectra of pristine cotton and sample A, sample B, and sample 23.

The FTIR spectra for pristine cotton, citric acid treated cotton, sample 23, sample A, and sample B are presented in Figure 5. The pristine cotton has an O-H stretching band at 3300 cm^−1^, which contains hydrogen bonding, and a band at 2900 cm^−1^ that is the result of C-H stretching. The band at 1310 cm^−1^ is due to C-H wagging, while the peak at 1640 cm^−1^ shows the presence of absorbed H_2_O molecules. The band at 1030 cm^−1^ is attributed to C=O stretching. The C–H bending is evident from the peak available at 1314 cm^−1^ [43,44]. After citric acid treatment, a new absorption peak appeared at 1729 cm^−1^, which can be attributed to the absorption of the carboxyl group from citric acid [45]. In the case of the treated samples, there are some new peaks. The peak due to P=O is centered at 1250 cm^−1^, while the peak centered at 884 cm^−1^ is associated with the P–O bond. Furthermore, the presence of the amide group can be confirmed by the peaks at 1624 cm^−1^ (amide vibration) and 1528 cm^−1^ (amide vibration), which are evidence of flame retardant treatment on the cotton fabric [46]. Moreover, in the case of sample A and sample 23, there is a major shift of FTIR spectra in the wavenumber range of 400 cm^−1^ to 500 cm^−1^, which can be attributed to the presence of ZnO NPs on the cotton fabric. In the case of sample 23, the intensity of spectra shift is more than sample A, which can be attributed to the high amount of ZnO NPs available in Sample 23. The shift of spectra in that wavenumber range can be attributed to the formation of the –CH_2_–O–Zn structure [41,47].

### 3.6. Thermal Stability

**Figure 6:**
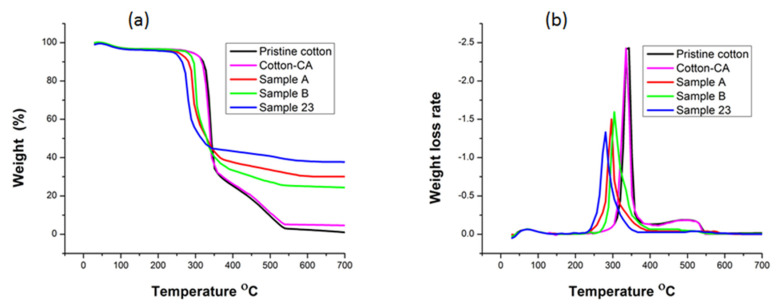
Thermo-oxidative behavior of (**a**) TGA curves and (**b**) dTG curves.

The thermal degradation trend of the fabric can be used to evaluate the flammability behavior of cotton fabric. Therefore, thermogravimetric analysis of pristine cotton and developed samples was performed in a synthetic air environment. Figure 6a show the weight loss percentage with the rise of temperature, Figure 6b show the weight loss rate with the rise of temperature, whereas Table 3 shows the values of decomposition temperatures for pristine cotton and developed samples. In the case of the TGA curve for pristine cotton, there is only little weight loss below 343 °C, which corresponds to the evaporation of water molecules. In this region, the decarboxylation and dehydration process of cotton cellulose occurred, forming the aliphatic char and combustible gasses. The region 350 °C to 550 °C corresponds to the transformation of aliphatic char into aromatic form carbon dioxide and carbon monoxide [48]. Citric acid-treated cotton fabric showed the same degradation behavior as pristine cotton but with little increase in residue. From Table 3, it can be seen that the T_onset_ 10% values shifted towards lower temperatures after MDPA and ZnO NPs coating. For pristine cotton, T_onset_ 10% is 319.23 °C, while for Sample 23, T_onset_ 10% is 266.07 °C which is the lowest of all the samples. This is attributed to the stronger performance of MDPA and ZnO NPs for the decomposition of cellulose as compared to pristine cotton. This T_onset_ mass loss is due to the evaporation of moisture contents from the fabric. Loading of MDPA and ZnO NPs tends to escalate the fabric’s moisture, hence lowering the T_onset_ for developed samples than pristine cotton [49]. T_max_ for pristine cotton was observed at 343.15 °C, while after treatment, T_max_ decreased, and the lowest T_max_ was observed for Sample 23 (i.e., 280.19 °C). From Table 3, it can be seen that the char residue at T_max_ and 600 °C increased after treatment compared to pristine cotton. This improvement can be explained as phosphorous components in MDPA were turned into phosphoric acid, which caused the fabric’s dehydration, hence leading to the lower degradation temperature and higher char residues [50]. The high char residual amount in the case of Sample 23 corresponds to ZnO NPs [51]. The quantitative amount of char residue produced is associated with flame retardance performance [52]. The reduction in degradation temperature after MDPA treatment might be due to the fact that the P–O–C bond is less stable than the C–O–C bond [53]. After ZnO NPs treatment, degradation temperature further decreased, which can be attributed to higher moisture contents in the fabric after ZnO NPs treatment [49]. The effect of ZnO NPs on the thermal stability can be described by coating theory; ZnO NPs formed a protective layer on the surface of the substrate, which restricted the reach of air to the substrate hence excluding the oxygen, finally affecting the thermal stability [51].

### 3.7. Vertical Flame Test

**Figure 7:**
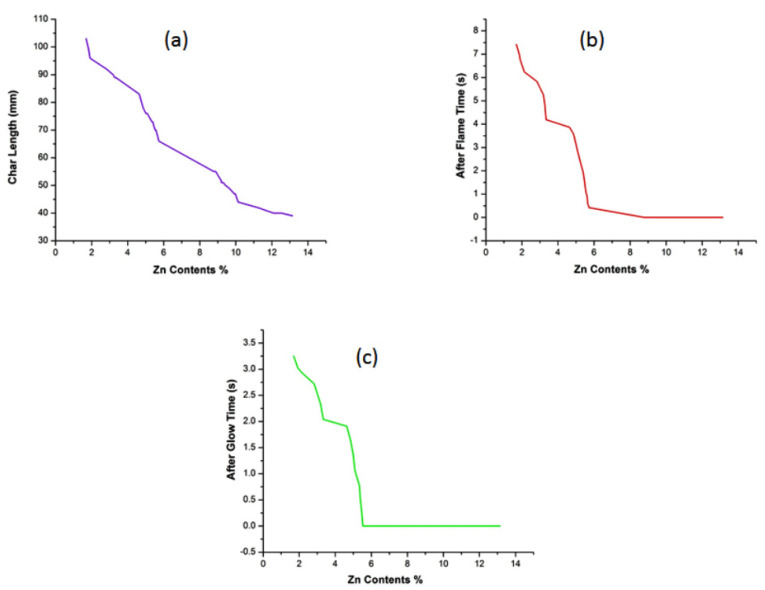
Flammability behavior of (**a**) char length against Zn contents, (**b**) after flame time against Zn contents, and (**c**) after glow time against Zn contents.

**Figure 8:**
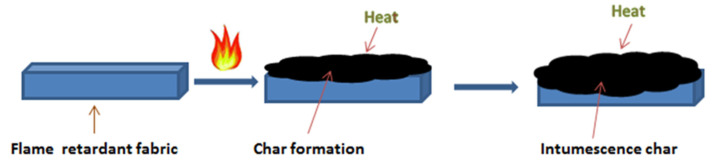
Mechanism of flame retardant fabric.

The measurements of the vertical flame test of untreated and developed samples are shown in Table 2 and Figure 7. It can be seen from Table 2 and Figure 7 that MDPA has a good effect on the flame retardancy of the cotton fabric, which is further improved by the deposition of ZnO NPs. It is evident from the results that flame retardant properties (i.e., after flame time, after glow time, and char length) improved with increased deposition of ZnO NPs. The untreated sample burned intensely in contact with flame. After detaching the flame source, the burning process of the untreated sample continued until it completely burned out without any char formation. On the other hand, all the treated samples (MDPA treated and MDPA + ZnO NPs treated) were self-extinguished. Furthermore, char formation was observed in the case of treated samples (MDPA treated and MDPA + ZnO NPs treated). Moreover, it was observed that the after flame time, after glow time, and char length of the treated samples decreased with an increased amount of Zn contents. The best flame retardant results were observed in the case of sonochemically optimized Sample 23. Sample 23 self-extinguished immediately after the removal of the combustion source and had zero seconds after flame time, zero seconds after glow time, and 39 mm char length. Sample A, developed by the conventional magnetic stirring method, had 2.13 seconds after flame time, zero seconds after glow time, and 76 mm char length, while sample B, only treated with MDPA, had 8.04 seconds after flame time, 5.21 seconds after glow time, and 127 mm char length. The char formation in the case of MDPA and MDPA + ZnO NPs treated samples was because of water removal from the fabric, which created the insulating layer and protected the fabric after flame removal, hence increasing the flame retardancy [54]. Furthermore, ZnO NPs acted as co-catalysts and decreased the flame spread rate; therefore, improved flame retardancy was achieved [55]. Figure 8 show the mechanism of flame retardancy. The comparison of current research results with literature is shown in Table 4.

### 3.8. Limiting Oxygen Index (LOI)

**Figure 9:**
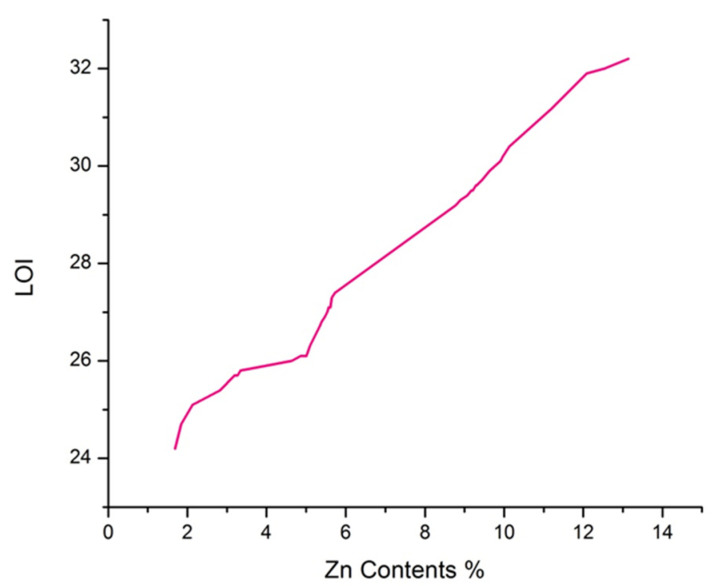
LOI values vs. Zn contents.

LOI can be defined as the minimum available percentage amount of oxygen gas in the oxygen/nitrogen gas mixture that is necessary to continue the combustion process of a material [60]. As the LOI value of a material is increased, it becomes more arduous to combustion. An LOI value of more than 27 indicates that the material is a flame retardant [60,61]. Table 2 and Figure 9 show the values for the LOI of treated and untreated fabric samples. It can be seen from Table 2 that the untreated sample has an LOI value of 17.6, which indicates that pristine cotton is highly combustible. On the other hand, sample B, having flame retardant application, has an LOI value of 23.8, which further increased after ZnO NPs application. Table 2 and Figure 9 show that the LOI value increased as the loaded concentration of ZnO NPs increased. These results are in accordance with Zhang et al., who concluded that the LOI value of cellulosic fibers increases as the loaded concentration of ZnO NPs increases [8]. The higher LOI value after ZnO NPs application might be due to the formation of a protective layer on fibers by ZnO NPs. The best LOI value was observed at 32.2 for sonochemical optimized sample 23, while sample A, developed by the conventional magnetic stirring method, had the LOI value of 27.7, which is very near to the LOI value (27.4) of sample 27, which had less ZnO NPs concentration compared to Sample A. This might be due to the homogenous and smooth distribution of ZnO NPs in the case of sample 27 after the sonochemical process compared to the conventional magnetic stirring process. The comparison of current research results with literature is shown in Table 5.

### 3.9. Antibacterial Activity

**Figure 10:**
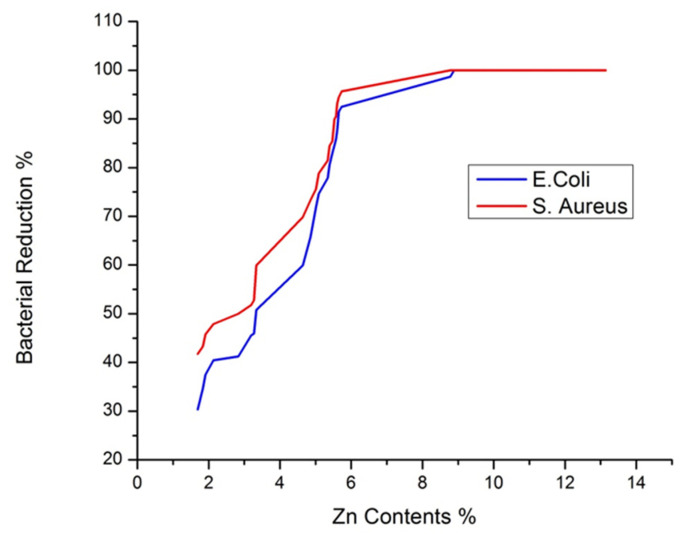
Bacterial reduction % vs. Zn contents.

Antibacterial activity of the developed samples was investigated according to the colony count test procedure and is shown in Table 2 and Figure 10. The results show that treated fabrics exhibit excellent bacterial reduction for both *E. coli* and *S. aureus* bacteria. From Table 2 and Figure 10, it is evident that with an increased loaded amount of ZnO NPs, the antibacterial activity of the treated samples also increased for both *E. coli* and *S. aureus* bacteria. Furthermore, 100% *S. aureus* reduction was achieved with an 8.78% loaded concentration of Zn contents (sample 17). In comparison, 100% *E. coli* reduction was achieved with a 9.07% loaded concentration of Zn contents (sample 18). As the ZnO NPs interact with bacteria, they generate reactive oxygen species, such as H_2_O_2_, •OH^−^, and •O_2_^−^. These reactive oxygen species damage the protein and DNA of the bacterial cell, resulting in the death of a bacterial cell. Furthermore, ZnO NPs deactivate the various necessary enzymes present in a bacterial cell; it is determined by the interaction between the ZnO NPs and the thiol group present in the bacterial cell. Moreover, the attachment of ZnO NPs onto the cell wall of the bacteria increases the concentration of the Zn^2+^ cations in the cytoplasm, which results in the death of bacteria [62,63,64]. A comparison of the current research results with the literature is presented in Table 6.

### 3.10. Ultraviolet Protection Factor (UPF)

There are three types of UV radiation in sunlight, i.e., UVA, UVB, and UVC. Among these radiations, UVA is the most dangerous; it harms human skin and is the main cause of DNA damage [67,68]. UV protective clothing can protect human skin from UV radiation. Ultraviolet protection factor (UPF) is one of the basic parameters to evaluate the UV-blocking ability of a fabric that specifies the potentiality of fabrics to shield the skin against UV radiation [69]. The Australian Standardization Institute classifies the protection level of fabric against its UPF value and the details are provided in Table 7 [70].

**Figure 11:**
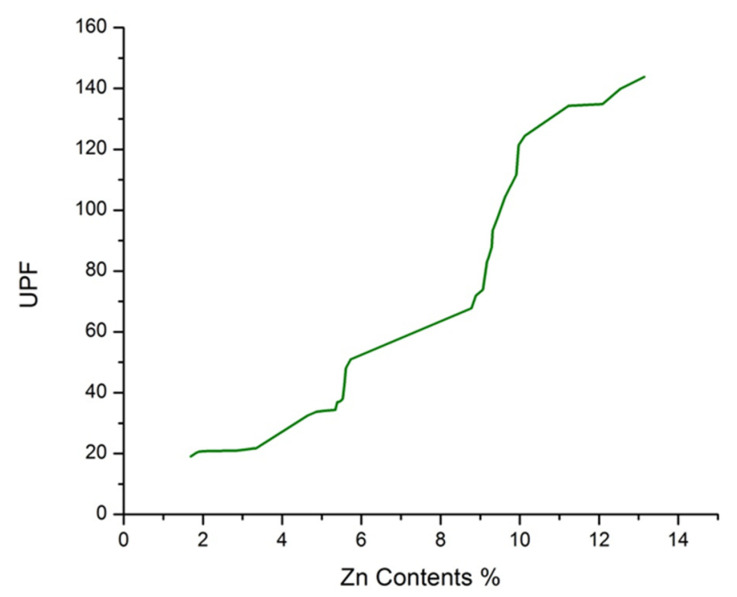
UPF values vs. Zn contents.

The UV protection factors (UPF values) of the untreated and developed samples are shown in Table 2 and Figure 11. It is apparent from Table 2 and Figure 11 that untreated cotton fabric has a UPF value of 4.78, while the UPF value of sonochemically synthesized optimized sample 23 has 143.76. It can also be seen from Table 2 and Figure 11 that with the increase in ZnO NPs concentration, the UPF values of the samples also increase. The study by Han and Yu supports these results; they concluded that the UV blocking ability of textile material increases with increasing metal oxide in the textile matrix [71]. The higher UPF value indicates that the fabric has a higher ability to protect against UV radiations [72]. ZnO NPs have a high refractive index, which causes UV radiations to be scattered when they interact with ZnO NPs, and not to be transmitted to the human body [73]. ZnO NPs also have a high ability to absorb UV radiations and convert them to infrared light, which is harmless [30,74]. Sample A with 7.83% ZnO NPs concentration has a 52.05 UPF value, and Sample 27 with 5.73% ZnO NPs has a 50.96 UPF value; the high difference in ZnO NPs concentrations and very little difference in UPF values of these samples can be explained as better and smooth distribution of ZnO NPs in the case of Sample 27 by the sonochemical process as compared to sample A which is prepared by the conventional stirring method. A comparison of the current research results with the literature is shown in Table 8.

### 3.11. Wash Durability

Table 9 show the results after 5, 10, and 20 wash cycles for Sample A, Sample B, and Sample 23. The results show that there is a gradual decrease in the Zn content, P content, flame retardancy, and functional properties of the sample after each wash cycle. However, in the case of ultrasonically optimized Sample 23, there is enough Zn and P content even after 20 wash cycles. Although char length increased to 52 mm and LOI decreased to 29.6 after 20 wash cycles for Sample 23, these values are excellent for flame retardancy. Sample 23 retained enough Zn content after 20 wash cycles and showed 100% bacterial reduction for both *S. aureus* and *E. coli* bacteria. Sample 23 showed an excellent UPF value of 123.16, even after 20 wash cycles.

## 4. Conclusions and Future Prospectives

In this research study, cotton fabric was modified by the ultrasonically-assisted in-situ synthesis of ZnO NPs and MDPA application by the conventional pad–dry–cure method. The study revealed that MDPA greatly affects the flame retardant performance of cotton fabric, which further increases by the deposition of ZnO NPs. For the deposition of ZnO NPs onto the cotton fabric, sonication time and concentrations of the chemical reagents were varied. The optimized conditions at 0.1 M zinc acetate, 0.3 M of NaOH, and 90 min of sonication time produced 13.14% Zn contents. The pure hexagonal wurtzite crystalline structure of ZnO NPs was confirmed by XRD. At optimal conditions, 22.4 nm crystallite sizes of ZnO NPs were observed. The grafting and presence of ZnO NPs were confirmed by ICP AES, FTIR, and SEM. The presence of phosphorous contents was confirmed by ICP AES, and grafting of phosphorous and amide group onto the cellulose structure was confirmed by FTIR. This research work disclosed that the concentration of ZnO NPs deposited onto the fabric has a direct correlation with flame retardancy and other functional properties. The optimized sample 23 showed excellent performance for flame retardancy before and after washing. Overall, 100% bacterial reduction for both *S. aureus* and *E. coli* bacteria was observed even after 20 wash cycles. The sample with the highest concentration of ZnO NPs showed a UPF value of 143.76 initially and 123.16 after 20 wash cycles.

Flame retardant multifunctional textiles at hand these days are the outcome of chemical treatments; at present, the technology that has been developed for producing flame retardant textiles based on nanomaterial is still at lab scale. The uses of nanoparticles impart some other desired properties. Future research should be focus attention on the application of nanoparticles as stuffing material, as their nano sizes allow them to penetrate into the interiors of polymer chains, hence imparting multifunctional properties. Along with ZnO NPs in the future, other metal oxide NPs (e.g., TiO_2,_ CuO, MgO etc.) should also be used in combination with MDPA to obtain the best FR/NPs system. Furthermore, the effect of the FR/NPs system on the fabric’s comfort properties (e.g., air permeability, moisture permeability, stiffness, heat transfer, etc.) should be studied. Moreover, there is a need to develop a statistical model to predict fabric’s functional properties for any given process parameter.

## Figures and Tables

**Table 1:** Molar concentrations of the precursors, sonication time, MDPA, resulting Zn contents, P contents, and add-on %.

Sample	Zinc Acetate (M)	NaOH (M)	Sonication Time (Minutes)	MDPA (g/L)	Zn Contents		P Contents		Add-On	
					(%)	Std. Dev.	(%)	Std. Dev.	(%)	Std. Dev.
1	0.05	0.1	30	300	1.69	0.071	3.88	0.083	16.92	0.157
2	0.05	0.1	60	300	1.84	0.041	3.86	0.079	17.11	0.128
3	0.05	0.1	90	300	2.13	0.093	3.83	0.062	17.45	0.152
4	0.05	0.1	120	300	1.91	0.080	3.84	0.074	17.22	0.161
5	0.05	0.2	30	300	2.83	0.107	3.81	0.086	18.31	0.186
6	0.05	0.2	60	300	3.19	0.099	3.79	0.053	18.61	0.148
7	0.05	0.2	90	300	3.34	0.138	3.78	0.094	18.91	0.226
8	0.05	0.2	120	300	3.27	0.071	3.79	0.062	18.78	0.142
9	0.05	0.3	30	300	4.64	0.108	3.75	0.057	20.38	0.171
10	0.05	0.3	60	300	4.86	0.067	3.73	0.082	20.70	0.132
11	0.05	0.3	90	300	5.09	0.103	3.72	0.065	20.96	0.155
12	0.05	0.3	120	300	5.01	0.055	3.72	0.068	20.84	0.133
13	0.1	0.1	30	300	5.34	0.073	3.71	0.054	21.23	0.123
14	0.1	0.1	60	300	5.47	0.085	3.70	0.073	21.43	0.163
15	0.1	0.1	90	300	5.65	0.051	3.70	0.064	21.60	0.127
16	0.1	0.1	120	300	5.53	0.054	3.70	0.062	21.51	0.112
17	0.1	0.2	30	300	8.78	0.077	3.63	0.076	25.32	0.168
18	0.1	0.2	60	300	9.07	0.067	3.61	0.058	25.65	0.143
19	0.1	0.2	90	300	9.31	0.043	3.58	0.050	25.48	0.194
20	0.1	0.2	120	300	9.17	0.071	3.60	0.074	25.77	0.203
21	0.1	0.3	30	300	11.23	0.064	3.50	0.049	28.24	0.162
22	0.1	0.3	60	300	12.09	0.076	3.47	0.038	29.21	0.181
23	0.1	0.3	90	300	13.14	0.068	3.44	0.067	30.47	0.129
24	0.1	0.3	120	300	12.54	0.059	3.46	0.052	29.79	0.117
25	0.15	0.1	30	300	5.39	0.059	3.72	0.068	21.31	0.134
26	0.15	0.1	60	300	5.61	0.068	3.69	0.048	21.54	0.108
27	0.15	0.1	90	300	5.73	0.042	3.69	0.056	21.72	0.112
28	0.15	0.1	120	300	5.57	0.054	3.70	0.072	21.52	0.138
29	0.15	0.2	30	300	8.89	0.050	3.62	0.058	25.42	0.116
30	0.15	0.2	60	300	9.21	0.024	3.59	0.037	25.82	0.089
31	0.15	0.2	90	300	9.43	0.041	3.55	0.048	26.11	0.102
32	0.15	0.2	120	300	9.29	0.032	3.58	0.064	25.91	0.113
33	0.15	0.3	30	300	9.63	0.064	3.54	0.028	26.31	0.094
34	0.15	0.3	60	300	9.91	0.034	3.53	0.038	26.65	0.097
35	0.15	0.3	90	300	10.13	0.051	3.52	0.058	26.91	0.124
36	0.15	0.3	120	300	9.97	0.079	3.53	0.046	26.57	0.146
A	0.1	0.3	90 (magnetic stirring)	300	7.83	0.102	3.67	0.061	24.09	0.188
B	-	-	-	300	-	-	3.92	0.077	14.93	0.93

**Table 2:** Experimental results for flammability test, LOI, bacterial reduction %, and UPF.

Sample	Flammability Test						LOI		Bacterial Reduction %				UV Protection	
	After Flame Time		After Glow Time		Char Length				*S. aureus*		*E. coli*			
	(s)	Std. Dev.	(s)	Std. Dev.	(mm)	Std. Dev.	(%)	Std. Dev.	R (%)	Std. Dev.	R (%)	Std. Dev.	UPF	Std. Dev.
Untreated	19.34	3.216	9.62	1.867	Completely burned	-	17.6	0.262	-	-	-	-	4.78	0.117
1	7.42	0.117	3.25	0.130	103	1.923	24.2	0.291	41.78	3.448	30.34	3.379	19.12	0.500
2	7.02	0.133	3.11	0.080	99	2.121	24.7	0.254	43.29	3.526	34.67	4.539	20.31	0.365
3	6.,24	0.176	2.94	0.107	95	1.224	25.1	0.071	47.86	3.720	40.45	3.423	20.87	0.254
4	6.74	0.119	3.03	0.084	96	0.707	24.8	0.187	45.76	4.211	37.47	4.502	20.64	0.145
5	5.83	0.212	2.72	0.175	92	2.000	25.4	0.141	49.97	5.535	41.23	5.051	20.94	0.333
6	5.26	0.168	2.33	0.167	90	2.345	25.7	0.100	51.79	4.442	45.57	3.760	21.53	0.390
7	4.19	0.222	2.04	0.074	89	0.704	25.8	0.158	59.93	3.761	50.78	4.039	21.72	0.289
8	4.84	0.253	2.17	0.137	89	1.000	25.7	0.072	52.79	5.963	45.91	2.483	21.67	0.354
9	3.87	0.224	1.91	0.113	83	1.870	26.0	0.122	69.86	4.751	59.92	3.621	32.52	0.418
10	3.58	0.178	1.62	0.077	78	3.114	26.1	0.212	73.32	3.729	65.76	3.305	33.71	0.294
11	2.82	0.204	1.06	0.059	76	1.581	26.3	0.108	78.84	4.300	74.65	2.734	34.13	0.206
12	3.12	0.147	1.34	0.123	76	1.214	26.1	0.123	75.54	4.326	71.78	2.152	33.98	0.214
13	2.09	0.213	0.78	0.092	73	1.225	26.7	0.119	81.45	3.413	77.87	2.160	34.39	0.231
14	1.56	0.167	0.27	0.054	71	1.870	26.9	0.164	85.42	4.032	82.98	3.313	37.17	0.376
15	0.59	0.108	0	0	68	2.645	27.3	0.137	94.43	1.606	91.46	4.328	49.09	1.586
16	1.17	0.125	0	0	70	2.549	27.0	0.094	89.95	2.142	84.56	2.688	37.98	0.906
17	0	0	0	0	55	2.738	29.2	0.146	100	0	98.64	0.869	67.74	1.103
18	0	0	0	0	53	2.121	29.4	0.086	100	0	100	0	73.89	1.623
19	0	0	0	0	51	2.236	29.6	0.092	100	0	100	0	93.34	2.465
20	0	0	0	0	52	2.915	29.5	0.128	100	0	100	0	82.98	2.613
21	0	0	0	0	42	2.167	31.2	0.114	100	0	100	0	134.32	3.181
22	0	0	0	0	40	1.788	31.9	0.099	100	0	100	0	134.87	2.776
23	0	0	0	0	39	0.707	32.2	0.102	100	0	100	0	143.76	3.439
24	0	0	0	0	40	1.581	32.0	0.110	100	0	100	0	139.93	2.645
25	1.97	0.145	0.53	0.036	73	1.140	26.8	0.146	84.49	2.597	80.54	2.172	36.89	0.581
26	0.92	0.115	0	0	69	2.726	27.0	0.173	93.23	2.100	87.76	2.338	48.04	1.034
27	0.42	0.078	0	0	65	1.643	27.4	0.085	95.67	1.378	92.51	2.311	50.96	1.452
28	1.02	0.106	0	0	70	2.126	27.1	0.167	90.42	2.404	85.78	1.636	42.74	1.215
29	0	0	0	0	55	2.166	29.3	0.118	100	0	100	0	71.87	1.092
30	0	0	0	0	51	1.789	29.5	0.090	100	0	100	0	84.45	1.467
31	0	0	0	0	50	1.562	29.7	0.114	100	0	100	0	97.12	3.991
32	0	0	0	0	51	3.741	29.6	0.158	100	0	100	0	87.92	2.032
33	0	0	0	0	49	1.303	29.9	0.172	100	0	100	0	104.45	2.279
34	0	0	0	0	47	0.836	30.1	0.126	100	0	100	0	111.56	3.003
35	0	0	0	0	44	1.870	30.4	0.132	100	0	100	0	124.47	5.080
36	0	0	0	0	47	1.224	30.2	0.121	100	0	100	0	121.34	2.711
A	2.13	0.754	0	0	76	3.824	27.7	0.192	96.27	7.358	93.52	5.674	52.05	6.092
B	8.04	0.246	5.21	0.232	127	4.949	23.8	0.097	-	-	-	-	13.23	0.268

**Table 3:** Thermal characteristics of pristine cotton and developed samples.

Sample	T_onset_ 10% (°C)	T_max_ (°C)	Residue at T_max_ (%)	Residue at 600 °C (%)
Pristine cotton	319.23	343.15	47.04	2.29
Cotton-CA	317.12	335.52	62.93	4.19
Sample A	280.34	296.13	68.47	30.91
Sample B	295.18	304.32	67.11	24.25
Sample 23	266.07	280.19	70.21	38.17

**Table 4:** Comparison of flame retardancy.

Fabric Treatment	After Flame Time (s)	After Glow Time (s)	Char Length (mm)	Reference
MDPA + ZnO NPs	0	0	39	This study
MDPA	0	0	59	[14]
MDPA + Dihydroxy ethylene urea	0.64	0	103	[56]
Diethyl methacryloylphosphoramidate	0	0	125	[57]
Bis(hydroxymethyl)phosphinic-methacrylate	0	0	90	[58]
Melamine salt of tannic phosphate	0	0	65	[59]

**Table 5:** Comparison of LOI.

Fabric Treatment	LOI	Reference
MDPA + ZnO NPs	32.2	This study
MDPA	26.3	[14]
Diethyl methacryloylphosphoramidate	30.2	[57]
Hydroxyl-functional organophosphorus	31.6	[52]
N,N-dimethylformamide + Zinc ion	30	[8]
MDPA + Dihydroxy ethylene urea	28.1	[56]

**Table 6:** Comparison of antibacterial activity.

ZnO NPs Synthesis Method	Bacterial Reduction %		Reference
	*S. aureus*	*E. coli*	
Sonochemical method	100	100	This study
Wet chemical method	>99.99	80	[65]
Microwave Irradiation Method	100	100	[23]
Solochemical process	100	-	[66]

**Table 7:** UPF value and protection category of fabric categorized by The Australian Standardization Institute.

UPF Value	Protection Level
Below 15	Not good
15–24	Good
24–39	Very good
40 and above	Excellent

**Table 8:** Comparison of UPF.

ZnO NPs Synthesis Method	UPF	Reference
Sonochemical Method	143.76	This study
One-step hydrothermal method	80.2	[31]
Two-step hydrothermal method	157.8	[35]
Microwave Irradiation Method	96.56	[23]

**Table 9:** Results of Zn contents, P contents, flammability test, LOI, bacterial reduction, and UPF after different wash cycles.

Sample	Zn Contents (%)	P Contents (%)	Flammability Test			LOI	Bacterial Reduction (%)		UPF
			After Flame Time (s)	After Glow Time (s)	Char Length (mm)		*S. aureus*	*E. coli*	
After 5 wash cycles									
Sample A	5.76	3.30	5.94	3.15	89	26.7	72.43	70.28	41.37
Sample B	-	3.48	10.32	5.19	134	22.1	-	-	11.81
Sample 23	11.38	3.11	0	0	46	30.3	100	100	132.92
After 10 wash cycles									
Sample A	4.74	3.13	7.03	3.52	93	24.9	63.23	60.96	34.87
Sample B	-	3.32	10.72	5.89	145	21.6	-	-	11.09
Sample 23	10.61	2.99	0	0	49	29.8	100	100	125.53
After 20 wash cycles									
Sample A	3.97	3.04	7.82	4.23	96	23.5	54.47	50.52	30.76
Sample B	-	3.24	11.29	6.08	149	20.4	-	-	10.61
Sample 23	10.17	2.93	0	0	52	29.6	100	100	123.16

## Data Availability

The data presented in this study are available on request from the corresponding author.
