# Correction: Javed et al. Zinc Oxide Nanoparticles (ZnO NPs) and N-Methylol Dimethyl Phosphonopropion Amide (MDPA) System for Flame Retardant Cotton Fabrics. *Polymers* 2022, *14*, 3414

**DOI:** 10.3390/polym15163337

**Published:** 2023-08-08

**Authors:** Asif Javed, Jakub Wiener, Jana Saskova, Jana Müllerová

**Affiliations:** 1 Department of Material Engineering, Faculty of Textile Engineering, Technical University of Liberec, Studentska 1402/2, 461 17 Liberec, Czech Republic; jakub.wiener@tul.cz (J.W.); jana.saskova@tul.cz (J.S.); 2 Department of Nanochemistry, Institute for Nanomaterials, Advanced Technologies and Innovation, Technical University of Liberec, Studentska 1402/2, 461 17 Liberec, Czech Republic; jana.mullerova@tul.cz

The authors wish to make a correction to this paper [1]. In the original publication, there was an error in defining the testing procedure of the nanoparticle size. A correction has been made in Section 2.5, paragraph 5. The revised version should be read as follows:

The particle size of the synthesized ZnO NPs was examined by employing dynamic light scattering (DLS) technology using the Malvern zeta sizer (Malvern Panalytical Ltd., Malvern, UK). After removing the fabric from the synthesis solution, the remaining solution was centrifuged at 5000 rpm for 3 min to separate the solid ZnO NPs from the liquid, and then the separated solid ZnO NPs were dried in an air oven at 90 °C for 120 min. The obtained ZnO NPs were dispersed in deionized water with the help of an ultrasonic probe. Eventually, the DLS technique was employed.

The authors wish to make the correction to Equation (9). The corrected equation is as follows:

(9) *LOI* = (100 × *O*_2_)/(*O*_2_ + *N*_2_)

The authors state that the scientific conclusions are unaffected. This correction was approved by the Academic Editor. The original publication has also been updated.
